# Imbalance Between Injury and Defense in the COPD Emphysematous Phenotype

**DOI:** 10.3389/fmed.2021.653332

**Published:** 2021-05-05

**Authors:** Shuang Bai, Li Zhao

**Affiliations:** Department of Pulmonary and Critical Care Medicine, Shengjing Hospital of China Medical University, Shenyang, China

**Keywords:** chronic obstructive pulmonary disease, emphysematous phenotype, pathogenesis, diagnosis, wound healing

## Abstract

The chronic obstructive pulmonary disease (COPD) emphysematous phenotype is characterized by destruction of lung tissue structure. Patients with this phenotype usually present with typical emphysema-like changes on chest computed Tomography CT, experience higher mortality and poorer prognosis, and are insensitive to routine pharmacological COPD therapy. However, the pathogenesis for the COPD emphysematous phenotype remains unclear, resulting in diagnostic and therapeutic challenges. The imbalance between injury and defense mechanisms is essential in the progression of many pulmonary diseases. Thus, in this review, we focus on the pathogenesis of the COPD emphysematous phenotype and discuss the pathophysiological processes involved in disease progression, from the perspective of injury and defense imbalance.

## Introduction

Chronic obstructive pulmonary disease (COPD) is a chronic respiratory disease that generally is manifested as cough, sputum, and breathlessness (1) with structural lung tissue damage and airway reconstitution (2). The clinical manifestations, medical imaging examinations, and pathologies of patients with COPD are quite heterogeneous (3, 4). In recent years, the definition of the COPD phenotype was proposed, and one of the most typical phenotypes is the emphysematous phenotype.

COPD patients with the emphysematous phenotype present with severe structural damage of lung tissue but slight airway destruction (5, 6). Emphysema in these patients deteriorates with disease progression (7), which affects respiration and exercise tolerance of patients. Patients suffer from several complications and experience an extremely high mortality rate (8, 9). There are few COPD therapies that are effective for the emphysema lesions of these patients, and specific pharmacological therapy does not exist. Therefore, delineating the exact pathogenic mechanisms of the emphysematous phenotype can achieve earlier diagnosis, precise categorization, and personalized therapeutic strategies, all of which are required to improve patient prognosis.

The imbalance between injury and defense mechanisms is essential for many pulmonary diseases, such as COPD, asthma, and pulmonary fibrosis. However, the role of the injury and defense system imbalance in the COPD emphysematous phenotype is still not fully understood. Previously, alpha antitrypsin (AAT) deficiency was defined as an injury-induced factor causing emphysema. However, it was found that a substantial number of COPD patients with emphysema present with normal AAT levels. This review illustrates the recent progress in COPD emphysematous phenotype pathogenesis, from an injury and defense imbalance perspective, and summarizes diagnostics and available therapeutic options to help improve COPD emphysematous phenotype prognosis.

## Manifestation and Pathophysiologic Processes of COPD Emphysematous Phenotype

The COPD phenotypes refer to disease characteristics that represent differences among COPD patients (10), including the chronic bronchitis phenotype, emphysematous phenotype, frequent exacerbators phenotype, and systemic inflammation phenotype. There are overlaps and interactions among these phenotypes (11). Patients with the emphysematous phenotype present a chronic pathophysiologic process with a decline of the elasticity of bronchioli terminals and distal airways and the destruction of lung tissue and alveolar walls, causing an overexpansion of distal airways and permanent enlargement in lung volume. As a result, COPD patients with an emphysematous phenotype present with reduced lung markings, lung volume enlargement, and decreased lung density (12).

Lung tissue damage and enhanced catabolism are the major pathophysiologic features of the COPD emphysematous phenotype. The restoring force of the lungs decreases as pulmonary elastic tissue is destroyed and insufficient pressure is applied to push air out during expiration. This results in the trapping of gas and excessive inflation. The motive range of the diaphragm narrows when the lungs expand, decreasing the blood volume returning to the heart. Thus, patients experience breathlessness and strain. Pulmonary ventilation dysfunction is also induced by the destruction of the air-blood barrier due to damaged alveoli and capillaries, resulting in air trapping and an imbalance of the ratio of ventilation and blood.

## Clinical Characteristics of Patients with the COPD Emphysematous Phenotype

Patients with the emphysematous phenotype usually show common symptoms of COPD, such as cough, expectoration, breathlessness, and more severe dyspnea. Some patients suffer both obvious dyspnea and poor exercise tolerance without airway inflammation-related symptoms. We analyzed chest CT scans, which illustrated unambiguous differences, resulting in the diversity of respiratory manifestations across patients. Moreover, various positions of emphysematous lesions affect pulmonary ventilation to a different extent. The observed respiratory symptoms were inconsistent with the results of the pulmonary function tests—the gold standard of COPD (13, 14). In this light, it is not difficult to infer that when the emphysema lesion doesn't affect the large airway, the spirometry of patients is likely to be normal. In this condition, although some smokers suffer from symptoms such as expiratory dyspnea, breathlessness, limitation of motion, and their condition is diagnosed as emphysema based on CT images; their condition cannot be diagnosed as COPD owing to their ratio of forced expiratory volume in 1 s to forced vital capacity (FEV1/FVC) being >70% (15, 16). Therefore, more attention should be dedicated to the emphysematous phenotype, even to the cases without airflow limitation, and the development of a suitable treatment strategy that is effective during the earlier stages can improve patient prognosis.

## Diagnostic Standards of COPD Emphysematous Phenotype

There is no uniform standard for the diagnosis of COPD patients with the emphysematous phenotype to date. The methods utilized in clinical studies can be divided into “spirometry-based classification” and “CT image-based classification.” The classifications of emphysematous phenotype analysis methods are shown in Table 1.

**Table 1:** Analysis methods of emphysematous phenotype judgement.

**Spirometry-based classification**				TLC > 20% pred, DLco < 80% pred
**CT image-based classification**	Density	“Density masking” method		
	Volume	Combined with thickness of layers and region of lung tissue		
	Destruction degree	Semi-quantitative evaluation	Goddard evaluation system	Score by emphysema area (involves each layer)
			Simplified Goddard evaluation system	Score by emphysema area (involves representative layers)
			Modified Goddard evaluation system	Score by emphysema area and emphysema degree
		Quantitative evaluation (proportion of the low density region)	LAA%	
			CT value thresholds	−910HU, −950HU, −960HU, or classify by CT value

### Spirometry-Based Classification

“Spirometry-based classification” is based on lung hyperinflation and impairment of lung diffusion capacity in COPD emphysematous phenotype. Cases of patients with total lung capacity (TLC) > 120% pred. (predicted value) and diffusion capacity for carbon monoxide in the lung (DLco) <80% pred. can be diagnosed as the emphysematous phenotype (17, 18). This classification method defines the COPD emphysematous phenotype from a qualitative perspective. However, the degree to which patients experience emphysema is ignored here. Diffusion measurements are taken into account, and this benefits the evaluation of the spirometry and respiratory symptoms.

### CT Image-Based Classification

With the advent of high-resolution CT, the spatial resolution of CT scan has been improved greatly, and the consistency between CT images and pathological sections are verified (12, 19). Therefore, “CT image-based classification” is the predominant method in use currently. However, there was no unified criterion for CT image-based classification methods because of the difference between CT parameters and subjects in every study.

In the quantitative assessment method, emphysematous lesions are mainly estimated from the density, volume, and degree of destruction in the lung tissue of patients with COPD (20–24). Lung density was calculated using the “density masking” method to calculate the ratio of area change (25). The evaluation of lung volume is based on continuous scanning images. The region of lung tissue in each scanning image is indicated by image analysis software. Then lung volume is obtained combined with the thickness of layers by calculating the total area of lung tissue (26).

The evaluation of the degree of destruction can be divided into semi-quantitative evaluation based on subjective observation and quantitative evaluation based on software. In 1982, Goddard proposed a semi-quantitative evaluation method named the Goddard evaluation system, widely used to assess the degree of destruction from emphysema (26, 27). The evaluation method was subsequently improved to fit specific purposes, based on the Goddard evaluation system. Some select the representative layers to analyze the degree of emphysema instead of involving all layers (22, 28). Others change the emphysema threshold (21) and include the emphysema region evaluation in their system (29, 30). Compared with the semi-quantitative evaluation based on subjective observation, quantitative evaluation based on software determines the emphysema phenotype by analyzing the proportion of low-density regions in the lung field. However, the CT value threshold used in analysis remains variable across users.

Previously, the −950HU and −910HU in the inspiratory phase were set up as the CT value thresholds in emphysema. There was a definition of the low attenuation area (LAA) %, which referred to the proportion of regions with CT values below the CT value threshold in the lung field. Patients with a LAA% below 20% would be diagnosed as having the emphysematous phenotype. Though the CT value thresholds differ, the difference of the variable CT value threshold did not affect the conclusion of the studies (31). Subsequently, some studies classify the emphysema degree by CT value threshold in the inspiratory phase (20, 32). CT value threshold utilized in other studies include −910HU (33), −950HU (34), and −960HU (35).

In addition to the variance across subjects and equipment parameters, the limitation of sample size is the main reason the CT value threshold is unsuitable for all patients. From an analysis of the relations between CT value thresholds and the emphysema index in related studies (31), it was demonstrated that the CT value in a certain pixel point was affected greatly, but the emphysema indexes were not changed significantly. This result indicated a suitable CT value can be chosen as the CT value threshold according to the actual condition. However, it is still essential to obtain the CT value of emphysema for the target population according to pathological specimens.

## Imbalance Between Injury and Defense Mechanism in the COPD Emphysematous Phenotype

**Figure 1:**
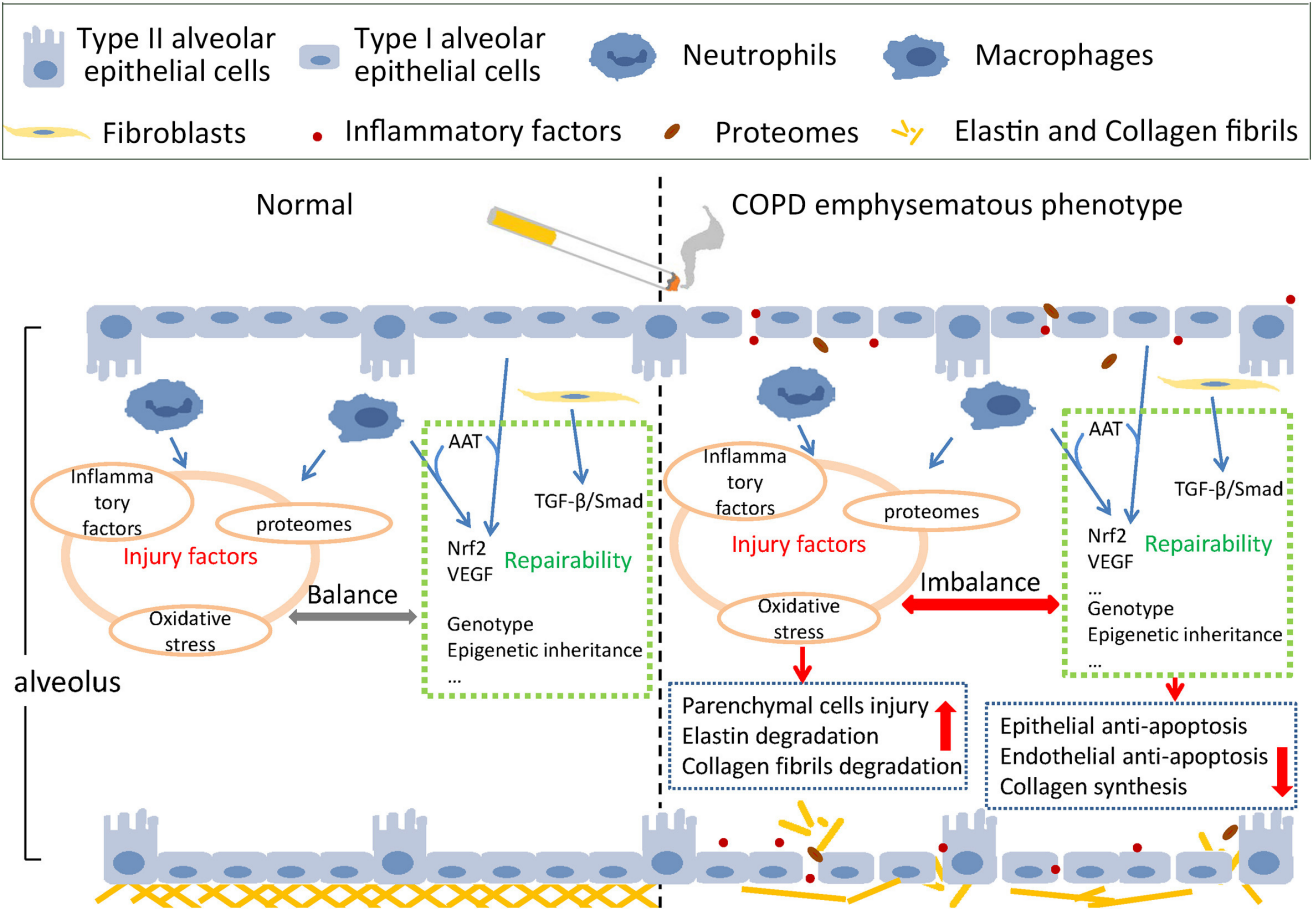
Schematic injury and defense imbalance in the COPD emphysematous phenotype. Cigarette smoke exposure induces injury enhancement and absence of defense repairability through various mechanisms and pathways, resulting in the destruction of alveolar structure and the development of emphysematous phenotype of chronic obstructive pulmonary disease.

The main pathological mechanism of emphysema includes protease activity enhancement, anti-protease activity reduction, oxidative stress, inflammation, apoptosis of alveolar epithelial and endothelial cells, tissue repair dysfunction, autophagy, the aging process, and necrosis (36), and most of these pathological processes were also related to the COPD emphysematous phenotype. We can summarize these pathological processes into two interactive aspects—injury factors and defense repairability of tissue (37). The schematic diagram is shown in Figure 1.

### Injury Factors

Usually, under the condition of stimulation, injury factors such as IL-6, IL-1β, and TNF-α are mainly produced by inflammatory cells and immune cells such as macrophages and neutrophils, which attack the lung parenchymal cells and mesenchymal components, including fibroblasts, elastin, and collagen fibrils. Many oxygen free radicals are produced, inducing severe inflammation and oxidative stress in alveolar epithelial cells and pulmonary vascular endothelial cells. Lung parenchymal cells experience uncontrolled apoptosis, and the pulmonary ventilation function is impaired (38, 39). At first, the AAT insufficiency was considered the main reason for emphysema occurrence in lungs (40). However, this hypothesis did not explain why smokers without AAT insufficiency still suffered from emphysema and the increase of neutrophil elastase (NE) was a typical feature for them (41). Accumulation of NE degrades elastin in lung mesenchyme causing irreversible damage to the mesenchymal structure.

The important role of matrix metalloproteinases (MMPs) was also identified in transgenic mice models. For example, knockout of MMP12 could rescue emphysema caused by smoking (42), even in mice with NE defect (43). Both inflammatory cells and immune cells, such as cytotoxicity T lymphocytes (CTLs) and natural killer (NK) cells, produce a type of proteinase—granzyme B (Gzm B)—under normal circumstances. Gzm B targets injury sites to clear abnormal cells, through perforin mediation. Once perforin deficient, excessive Gzm B degrades the extracellular matrix, destroys elastin and collagen, and causes severe lung damage (44).

All pathological processes mentioned above induce the imbalance of proteinase and antiprotease and degradation of elastin and collagen fibrils. Once the lung mesenchyme is destroyed by excessive injury factors, the lung tissue dynamic compliance is attenuated, and the alveolus inflates, eventually leading to emphysema.

### Defense Repairability

Defense repairability of lung parenchymal cells mainly involves the anti-apoptotic effects of alveolar epithelial cells and the “stem-like” repair function of type II alveolar epithelial cells. Type I and Type II alveolar epithelial cells, vascular endothelial cells, and myofibroblasts make up the alveolar interval. When cigarette smoke acts on the alveolar interval, cellular immunity is activated, and CD8+ T cells react immediately, resulting in epithelial cell apoptosis (45). Vascular endothelial growth factor (VEGF) is secreted by epithelial cells and macrophages and acts on pulmonary vascular endothelial cells to resist apoptosis. Nuclear Factor, Erythroid 2 Like 2 (NFE2L2/Nrf2), one of the most important transcription factors of antioxidant reaction, combines with AAT and VEGF to work together in the antiapoptotic process (41). AAT inhibits apoptosis of epithelial and vascular endothelial cells by interacting directly with cleaved-caspase3 (39, 46).

Under normal conditions, type II alveolar epithelial cells are responsible for secreting alveolar surfactant, sustaining tension and dynamic compliance, and regulating inflammation and cytokines (47). When alveolar epithelial cells are damaged, type II alveolar epithelial cells differentiate into type I alveolar epithelial cells to repair the alveolar structure. The destruction of type II alveolar epithelial cells affects lung parenchymal structure and pulmonary function. Mesenchymal cells, specifically fibroblasts, play an important role during the complicated process of lung mesenchyme repair (48–51). When lung mesenchyme hurts, elastases hydrolyze elastin, and collagen contraction is inhibited. Fibroblasts repair collagen through *de novo* collagen synthesis (52–54). The dysfunction-related pathways of fibroblasts were also examined through bioinformatic analysis of emphysema transcriptomics (55). As macrophages and other inflammatory cells accumulate around emphysema lesions and small airways, inflammatory factors and proteases are secreted, resulting in inflammation and mesenchymal degradation. TGF-β combines with its receptors and phosphorylates Smad2/3 by cascade reactions to activate the repairing-related pathways and improve the synthesis of fibrin in fibroblasts (56). The activity of TGF-β pathways is spatially different in the lungs. Smad2 phosphorylation decreased in emphysematous lesions, whereas it increased around small airways, consistent with the pathological manifestation of COPD patients with emphysema—alveolar destruction and thickening of the small airway mesenchyme (57).

Members of the lysamyl oxidase (LOX) superfamily also play an important role in the repair mechanism of emphysema. Specific amino acid residues on collagen and elastin can be oxidized by LOX in the extracellular matrix, forming covalent bonds between collagen and elastin, with the participation of Cu2+ (58–62). Single-nucleotide polymorphisms (SNP), such as rs1828591 and rs13118928 on the gene of Hedgehog Interacting Protein (HHIP), are intensively related to COPD (63). HHIP negatively regulates cell proliferation and matrix repair under normal conditions (64). By comparing mesenchymal cells in the lung tissue of emphysema and normal subjects through single-cell sequencing, it was found that the Hh signaling pathways were atopically activated in the alveolar matrix, impairing alveolar stem cells. Therefore, it is speculated that the SNP of HHIP facilitates the formation of emphysema by functional incapacitation (65). In addition to the influence of genotype on COPD emphysema phenotype, epigenetic inheritance also plays an important role. Some studies have documented that cigarette smoke can change the expression of COPD-related genes by DNA methylation and posttranslational modification of histones.

In brief, inflammatory factors and proteinases multiply and damage pulmonary structural cells; matrix components are degraded, and alveolar cell apoptosis is induced. The repair and regeneration of parenchymal and interstitial cells do not compensate for the impaired pulmonary tissue. Eventually, all the pulmonary tissue gets structurally damaged, enabling emphysema formation (38, 65, 66).

## Conclusion

The COPD emphysematous phenotype is characterized by specific manifestations and has a high mortality rate. The injury and defense mechanisms play important roles in the occurrence and development of this phenotype. Thus, a more in-depth understanding of the related radiological and mechanistic features to define a suitable CT value threshold and create personalized therapies is warranted. This can enable improved diagnostic and treatment options that improve patient prognosis in those affected with the COPD emphysematous phenotype.

## Author Contributions

LZ substantially contributed to the conception and design of the manuscript. SB and LZ contributed to the acquisition, analysis, interpretation of data, drafted the article, revised it critically and substantially, have approved the final version, and accepted accountability for all aspects of the study. All authors contributed to the article and approved the submitted version.

## Conflict of Interest

The authors declare that the research was conducted in the absence of any commercial or financial relationships that could be construed as a potential conflict of interest.
